# Diagnostic value of tumor markers in identifying favorable or unfavorable subsets in patients with cancer of unknown primary: a retrospective study

**DOI:** 10.1186/s12885-022-09514-3

**Published:** 2022-04-14

**Authors:** Shigemasa Takamizawa, Tatsunori Shimoi, Masayuki Yoshida, Momoko Tokura, Shu Yazaki, Chiharu Mizoguchi, Ayumi Saito, Shosuke Kita, Kasumi Yamamoto, Yuki Kojima, Hitomi Sumiyoshi-Okuma, Tadaaki Nishikawa, Emi Noguchi, Kazuki Sudo, Kan Yonemori

**Affiliations:** 1 Department of Medical Oncology, National Cancer Center Hospital, 5-1-1 Tsukiji, Chuo-Ku, Tokyo, 104-0045 Japan; 2 Department of Diagnostic Pathology, National Cancer Center Hospital, 5-1-1 Tsukiji, Chuo-Ku, Tokyo, 104-0045 Japan

**Keywords:** Cancer of unknown primary, Favorable subsets, NCC-ST-439 (ST439), Sialyl-Tn (STN), Tumor marker, Unfavorable subsets

## Abstract

**Background:**

Routine measurement of tumor markers is not recommended in daily clinical practice for patients with cancer of unknown primary (CUP). We evaluated the diagnostic value of tumor markers in identifying favorable or unfavorable subsets in patients with CUP.

**Methods:**

We retrospectively reviewed the medical records of patients who were diagnosed with CUP between October 2010 and July 2015 at the National Cancer Center Hospital. The tumor markers of the patients were examined, including squamous cell carcinoma antigen, cytokeratin fraction, carcinoembryonic antigen, sialyl Lewis X, neuron-specific enolase, pro-gastrin-releasing peptide, α-fetoprotein, protein induced by vitamin K absence or antagonist II, prostate-specific antigen, soluble interleukin-2 receptor, carbohydrate antigen 19–9, cancer antigen 125, cancer antigen 15–3, NCC-ST-439 (ST439), elastase-1, human chorionic gonadotropin, and sialyl-Tn (STN).

**Results:**

Among 199 patients with suspected CUP, 90 were diagnosed with confirmed CUP (12 in the favorable subset and 78 in the unfavorable subset). No tumor markers showed 100% sensitivity for unfavorable subsets. ST439 (*p* = 0.03) and STN (*p* = 0.049) showed 100% specificity for unfavorable subsets.

**Conclusions:**

For patients with suspected CUP who show elevated ST439 or STN levels, the treatment strategy should be based on the premise that the patient is likely to be placed in the unfavorable subset.

## Background

Cancer of unknown primary (CUP) is defined as a cancer lacking any detectable primary site after full evaluation. Only metastatic sites are histologically confirmed. CUP is a rare malignancy, accounting for approximately 3%–5% of all newly diagnosed patients with malignancies [1]. In addition, some are found to be non-cancerous during a thorough examination [2]. Approximately 20% of patients with CUP have a favorable prognosis [1]. This patient group includes men with adenocarcinoma of bone metastases and elevated prostate-specific antigen (PSA), women with papillary adenocarcinoma of the peritoneal cavity, women with adenocarcinoma involving the axillary lymph nodes, patients with poorly differentiated carcinoma with midline distribution, patients with well-differentiated neuroendocrine tumors or poorly differentiated neuroendocrine carcinomas, patients with squamous cell carcinoma involving cervical lymph nodes, patients with adenocarcinoma with a colon cancer profile, and patients with squamous cell carcinoma of isolated inguinal adenopathy [1]. These patients should be identified at initial evaluation and receive specific therapy to extend the prognosis.

Approximately 80% of patients with CUP do not have any favorable subsets, and the prognosis of these patients is worse [1]. The median survival time of these patients is only 6–7 months [1], and a standard of care for this patient group is absent [3]. Although most patients with unfavorable subsets are treated based on suspected tissue-of-origin, there is no survival advantage compared with empiric platinum-based combination chemotherapy [4]. A previous report analyzed 93 patients who received platinum-based combination chemotherapy, and the response rate was 39.8% [5]. A meta-analysis has shown that no type of chemotherapy has been proven to lengthen survival time [6].

Evaluation of tumor markers is useful for diagnosis and the reduction of inappropriate diagnostic tests for patients with suspected malignancy [7]. Although patients with CUP commonly overexpress several tumor markers, the diagnostic, predictive, and prognostic utilities are unexplained. Routine measurement of tumor markers for patients with CUP is not recommended in daily clinical practice [8].

Tumor markers are not recommended for finding the primary site of CUP, except in limited situations. The guidelines published by the European Society for Medical Oncology mention that useful tumor markers for diagnosing the primary tumor site include human chorionic gonadotropin (hCG) and α-fetoprotein (AFP) in patients with poorly differentiated carcinoma of midline distribution for germ-cell tumors, PSA in men with bone metastases for prostate cancer, cancer antigen 125 (CA125) in women with primary peritoneal serous adenocarcinoma for ovarian, fallopian tube, and peritoneal cancers, and thyroglobulin for differentiated thyroid cancer [1, 8–13].

Squamous cell carcinoma antigen is a marker that is elevated in squamous cell carcinomas, such as head and neck, esophageal, and uterine cervical cancers [14]. Cytokeratin fraction (cytokeratin 19 fragment) is elevated in non-small cell lung cancers [15]. Carcinoembryonic antigen, present in the fetal digestive cells, is elevated in gastric, colorectal, and other cancers of the digestive system [16]. Sialyl Lewis X is a polymeric glycoprotein elevated in lung, ovarian, and pancreatic cancers [17]. Neuron-specific enolase increases with the tumorigenesis in neuroendocrine cells, such as in small-cell lung cancer and neuroblastoma [18]. Pro-gastrin-releasing peptide is a gastrointestinal hormone; it is a marker for small-cell lung cancer [19]. Protein induced by vitamin K absence or antagonist II is precursor of the coagulation factor prothrombin; it is a marker for hepatocellular carcinoma [20]. Soluble interleukin-2 receptor is the alpha chain of interleukin 2 receptor, which exists in the free-form in blood; it is elevated in lymphoid malignancies such as non-Hodgkin's lymphoma, adult T-cell lymphoma/leukemia, and acute lymphocytic leukemia [21]. Carbohydrate antigen 19–9 is a cell surface glycoprotein complex; it is elevated in gastrointestinal cancers, such as pancreatic, gallbladder, and bile duct cancers [22]. Cancer antigen 15–3 is a mucin-type glycoprotein, which is elevated in breast cancers [23]. NCC-ST 439 is a mucin-type glycoprotein that is elevated in breast and gastrointestinal cancers [24]. Elastase-1 is a proteolytic enzyme; it is a marker for pancreatic cancer [25]. Sialyl-Tn is a sugar chain antigen; it is elevated in ovarian and gastrointestinal cancers [26]. However, these tumor markers are not recommended for identifying the primary site of CUP.

The diagnostic evaluation of patients with CUP takes time, sometimes up to several months. In addition, deciding whether a subset is favorable or not must be carefully considered because it has a great impact on the treatment selection and prognosis. Only a few studies have examined whether tumor markers can be used to classify subsets. If tumor markers could be used to classify favorable and unfavorable subsets, then the best treatment option could be more quickly recommended to patients with CUP. Identifying favorable subsets during the initial evaluation can lead to appropriate treatment and prolonged survival. We evaluated the diagnostic value of tumor markers that are routinely used in our hospital for identifying favorable or unfavorable subsets in patients with CUP.

## Methods

### Study cohort

We retrospectively reviewed the medical records of patients who were diagnosed with CUP at National Cancer Center Hospital (NCCH) (Tokyo, Japan) between October 2010 and July 2015. This single-institution medical record-based retrospective observational study was approved by the Institutional Review Board of NCCH (NCCH 2012–335), which waived the requirement for informed consent. Patient registration was based on an opt-out model. The study was conducted according to the principles of the Declaration of Helsinki.

### Diagnosis of CUP

Since our facility is a cancer-specialized hospital, most patients with suspected cancer were referred to us before they had undergone adequate examination. Patients then underwent a fundamental workup and additional focused imaging based on their cancer distribution and histopathology. Examinations were performed according to the guidelines of the European Society for Medical Oncology and the Japanese Society of Medical Oncology [12].

Patients were evaluated through an initial workup, including physical examination, laboratory studies (a complete blood count, urinalysis, basic serum chemistries, and tumor marker analysis), and imaging procedures (computed tomography scan or magnetic resonance imaging (MRI) of the chest, abdomen, and pelvis). The selected women were evaluated with a pelvic examination by a gynecologist or mammography and breast MRIs to look for breast lesions. For selected men or women, an examination of the prostate or urinary tract by a urologist was completed to look for urinary tract lesions.

The diagnosis of CUP was confirmed when the primary site of cancer was unknown after these initial workups, based on the consensus of medical oncology specialists.

### Tumor markers

The patients were evaluated for the following tumor markers: squamous cell carcinoma antigen (cut-off: 1.5 ng/ml), cytokeratin 19 fragment (cut-off: 2.2 ng/ml), carcinoembryonic antigen (cut-off: 5.0 ng/ml), sialyl Lewis X (cut-off: 38.0 U/ml), neuron-specific enolase (cut-off: 15.0 ng/ml), pro-gastrin-releasing peptide (cut-off: 81.0 pg/ml), AFP (cut-off: 10.0 ng/ml), protein induced by vitamin K absence or antagonist II (PIVKA-II) (cut-off: 40 mAU/ml), PSA (cut-off: 2.7 ng/ml), soluble interleukin-2 receptor (sIL-2R) (cut-off: 587 U/ml), carbohydrate antigen 19–9 (cut-off: 37 U/ml), CA125 (cut-off: 35 U/ml), cancer antigen 15–3 (cut-off: 28 U/ml), NCC-ST 439 (ST439) (cut-off: 4.5 U/ml), elastase-1 (cut-off: 300 ng/dl), hCG (cut-off: 3.0 mIU/ml), and sialyl-Tn (STN) (cut-off: 45.0 U/ml). These cut-off values were based on the facility standard.

### Definition of favorable and unfavorable subsets

The following patient populations were placed in the favorable subset: men with adenocarcinoma who have bone metastases and elevated PSA; women with adenocarcinoma who have peritoneal carcinomatosis; women with adenocarcinoma who have axillary lymph node metastases; patients with poorly differentiated carcinoma with midline distribution; patients with well-differentiated neuroendocrine tumors or poorly differentiated neuroendocrine carcinomas; patients with squamous cell carcinoma involving the cervical nodes; patients with a colon cancer profile; and patients with squamous cell carcinoma of isolated inguinal adenopathy [12, 13]. Patients without any of these factors were placed in the unfavorable subset.

### Statistical analyses

Univariate analyses were performed to evaluate the correlation between tumor markers and favorable or unfavorable subsets. All statistical analyses were performed using JMP software (version 14.3.0 for Windows; SAS Institute Japan Inc., Cary, NC, USA), and results were considered significant with a two-sided *p*-value of < 0.05.

## Results

### Patient characteristics

**Fig. 1:**
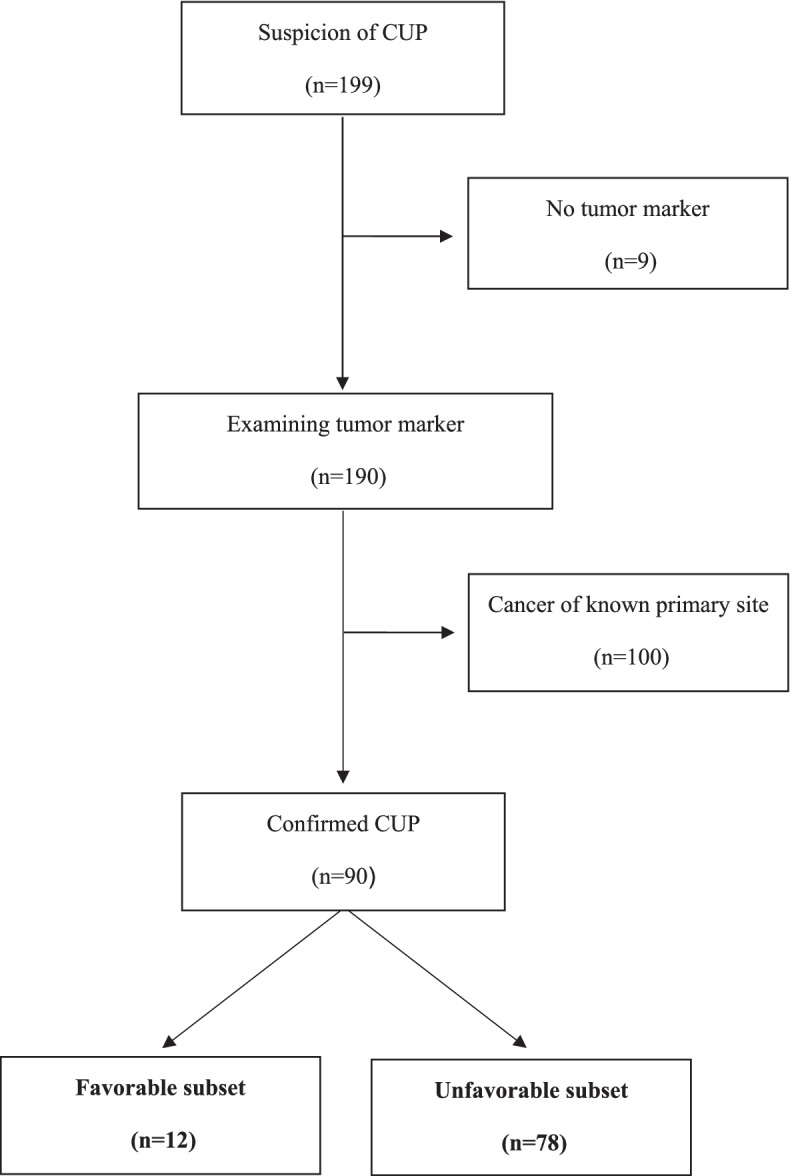
CONSORT diagram of patient selection

Between October 2010 and July 2015, 199 patients with suspected CUP were referred to the NCCH. Among them, 190 patients were examined via tumor markers, 100 were diagnosed with cancer of known primary site, and 90 were diagnosed with confirmed CUP (12 in the favorable subset and 78 in the unfavorable subset) (Fig. 1).

Median age was 68 years (range: 47–78) in the favorable subset and 66 years (range: 34–83) in the unfavorable subset. Females accounted for 83% of the favorable subset and 55% of the unfavorable subset. The Eastern Cooperative Oncology Group Performance Status (ECOG-PS) of most patients was 0 or 1. The estimated primary organs of 12 patients in the favorable subset were breast (5 patients), ovary/peritoneum (5 patients), and skin (2 patients). The characteristics of the patients are shown in Table 1.

**Table 1:** Patient characteristics

		Favorable (*n* = 12)	Unfavorable (*n* = 78)	Total (*n* = 90)
Age in years	Median (range)	68 (47–78)	66 (34–83)	66.5 (34–83)
Sex, n (%)	Male	2 (17)	35 (45)	37 (41)
	Female	10 (83)	43 (55)	53 (59)
ECOG-PS, n (%)	0	9 (75)	36 (46)	45 (50)
	1	2 (17)	33 (42)	35 (39)
	2	1 (8)	2 (3)	3 (3)
	3	0	6 (8)	6 (7)
	NA	0	1 (1)	1 (1)
Metastases, n (%)	Lymph node	5 (42)	53 (68)	58 (64)
	Bone	0	21 (27)	21 (23)
	Liver	0	13 (17)	13 (14)
	Lung	0	12 (15)	12 (13)
	Brain	0	4 (5)	4 (4)
The estimated primary organ, n (%)	Breast	5 (42)	-	-
	Ovary/peritoneum	5 (42)	-	-
	Skin	2 (17)	-	-

*ECOG* Eastern Cooperative Oncology Group, *NA* not applicable, *PS* performance status

### Sensitivity and specificity of tumor markers

No tumor markers showed 100% sensitivity for unfavorable subsets. However, PIVKA-II, PSA, ST439, elastase-1, and STN showed 100% specificity for unfavorable subsets. Among them, ST439 (*p* = 0.03) and STN (*p* = 0.049) showed a significant correlation between favorable and unfavorable subsets (Table 2).

**Table 2:** Sensitivity and specificity of unfavorable subsets for each tumor marker

		Favorable (*n* = 12)	Unfavorable (*n* = 78)	*p*	Sensitivity of Unfavorable	Specificity of Unfavorable
SCC, n (%)	≤ 1.5 ng/ml	8 (67)	63 (81)	0.65	15%	80%
	> 1.5 ng/ml	2 (17)	11 (14)			
	NA	2 (17)	4 (5)		-	-
CYFRA, n (%)	≤ 2.2 ng/ml	5 (42)	23 (30)	0.29	69%	50%
	> 2.2 ng/ml	5 (42)	52 (67)			
	NA	2 (17)	3 (4)		-	-
CEA, n (%)	≤ 5.0 ng/ml	10 (83)	46 (59)	0.12	41%	83%
	> 5.0 ng/ml	2 (17)	32 (41)			
SLeX, n (%)	≤ 38.0 U/ml	6 (50)	49 (63)	1.0	34%	67%
	> 38.0 U/ml	3 (25)	25 (32)			
	NA	3 (25)	4 (5)		-	-
NSE, n (%)	≤ 15.0 ng/ml	8 (67)	41 (53)	0.18	45%	80%
	> 15.0 ng/ml	2 (17)	33 (42)			
	NA	2 (17)	4 (5)		-	-
Pro-GRP, n (%)	< 81.0 pg/ml	9 (75)	66 (85)	1.0	11%	90%
	≥ 81.0 pg/ml	1 (8)	8 (10)			
	NA	2 (17)	4 (5)		-	-
AFP, n (%)	≤ 10.0 ng/ml	8 (67)	68 (87)	0.24	8%	80%
	> 10.0 ng/ml	2 (17)	6 (8)			
	NA	2 (17)	4 (5)		-	-
PIVKA-II, n (%)	< 40 mAU/ml	10 (83)	70 (90)	1.0	4%	100%
	≥ 40 mAU/ml	0	3 (4)			
	NA	2 (17)	5 (6)		-	-
PSA, n (%)	≤ 2.7 ng/ml	3 (25)	29 (37)	1.0	22%	100%
	> 2.7 ng/ml	0	8 (10)			
	NA	9 (75)	41 (53)		-	-
sIL-2R, n (%)	≤ 587 U/ml	0	3 (4)	-	40%	-
	> 587 U/ml	0	2 (3)			
	NA	12 (100)	73 (94)		-	-
CA19-9, n (%)	≤ 37 U/ml	8 (67)	56 (72)	1.0	27%	80%
	> 37 U/ml	2 (17)	21 (27)			
	NA	2 (17)	1 (1)		-	-
CA125, n (%)	≤ 35 U/ml	6 (50)	26 (33)	0.32	63%	55%
	> 35 U/ml	5 (42)	45 (58)			
	NA	1 (8)	7 (9)		-	-
CA15-3, n (%)	≤ 28 U/ml	9 (75)	40 (51)	0.22	46%	75%
	> 28 U/ml	3 (25)	34 (44)			
	NA	0	4 (5)		-	-
ST439, n (%)	≤ 4.5 U/ml	11 (92)	50 (64)	**0.03**	32%	100%
	> 4.5 U/ml	0	24 (31)			
	NA	1 (8)	4 (5)		-	-
elastase-1, n (%)	≤ 300 ng/dl	10 (83)	68 (87)	1.0	7%	100%
	> 300 ng/dl	0	5 (6)			
	NA	2 (17)	5 (6)		-	-
hCG, n (%)	≤ 3.0 mIU/ml	8 (67)	64 (82)	0.62	12%	80%
	> 3.0 mIU/ml	2 (17)	9 (12)			
	NA	2 (17)	5 (6)		-	-
STN, n (%)	≤ 45.0 U/ml	8 (67)	46 (59)	**0.049**	36%	100%
	> 45.0 U/ml	0	26 (33)			
	NA	4 (33)	6 (8)		-	-

*AFP* α-fetoprotein, *CA15-3* Cancer antigen 15–3, *CA19-9* Carbohydrate antigen 19–9, *CA125* Cancer antigen 125, *CEA* Carcinoembryonic antigen, *CYFRA* Cytokeratin fraction, *hCG* human chorionic gonadotropin; *NA* Not applicable, *NSE* Neuron-specific enolase, *p* P-value, *PIVKA-II* Protein induced by vitamin K absence or antagonist II, *Pro-GRP* Pro-gastrin-releasing peptide, *PSA* Prostate-specific antigen, *SCC* Squamous cell carcinoma antigen, *sIL-2R* Soluble interleukin-2 receptor, *SLeX* Sialyl Lewis X, ST439: NCC-ST 439; *STN*, Sialyl-Tn

Results were considered significant with a two-sided *p*-value of < 0.05

### Treatment regimen

Among the patients placed in the unfavorable subset, many were treated with drug therapy based on suspected tissue-of-origin, such as lung, based on the consensus of medical oncologists (Table 3 and 4). Patients within the normal range of ST439 were significantly more likely to receive drug therapy for ovarian cancer (*p* = 0.004). Patients with elevated ST439 levels were significantly more likely to receive drug therapy for colorectal (*p* = 0.036) or salivary gland cancer (*p* = 0.031).

**Table 3:** Treatment regimen for patients with unfavorable subsets according to ST439

ST439	≤ 4.5 U/ml (*n* = 50)	> 4.5 U/ml (*n* = 24)	Total (*n* = 74)	p
Lung, n (%)	13 (26)	6 (25)	19 (26)	1.0
Ovarian/Peritoneal, n (%)	17 (34)	1 (4)	18 (24)	**0.004**
CUP, n (%)	9 (18)	4 (17)	13 (18)	1.0
Breast, n (%)	4 (8)	2 (8)	6 (8)	1.0
Colorectal, n (%)	1 (2)	4 (17)	5 (7)	**0.036**
Ureteral/Kidney, n (%)	2 (4)	1 (4)	3 (4)	1.0
Biliary tract, n (%)	1 (2)	2 (8)	3 (4)	0.24
Salivary gland, n (%)	0	3 (13)	3 (4)	**0.031**
Cervical, n (%)	0	1 (4)	1 (1)	0.32
Nasopharyngeal, n (%)	1 (2)	0	1 (1)	1.0
Esophageal, n (%)	1 (2)	0	1 (1)	1.0
Skin, n (%)	1 (2)	0	1 (1)	1.0

*CUP* Cancer of unknown primary, *p* P-value; ST439: NCC-ST 439

Results were considered significant with a two-sided *p*-value of < 0.05

**Table 4:** Treatment regimen for patients with unfavorable subsets according to STN

STN	≤ 45.0 U/ml (*n* = 46)	> 45.0 U/ml (*n* = 26)	Total (*n* = 72)	p
Lung, n (%)	15 (33)	4 (15)	19 (26)	0.16
Ovarian/Peritoneal, n (%)	9 (20)	9 (35)	18 (25)	0.17
CUP, n (%)	5 (11)	5 (19)	10 (14)	0.48
Breast, n (%)	5 (11)	1 (4)	6 (8)	0.41
Colorectal, n (%)	2 (4)	3 (12)	5 (7)	0.34
Ureteral/Kidney, n (%)	3 (7)	1 (4)	4 (6)	1.0
Biliary tract, n (%)	2 (4)	1 (4)	3 (4)	1.0
Salivary gland, n (%)	1 (2)	2 (8)	3 (4)	0.29
Cervical, n (%)	1 (2)	0	1 (1)	1.0
Nasopharyngeal, n (%)	1 (2)	0	1 (1)	1.0
Esophageal, n (%)	1 (2)	0	1 (1)	1.0
Skin, n (%)	1 (2)	0	1 (1)	1.0

*CUP* cancer of unknown primary, *p*: P-value, *STN* Sialyl-Tn

Results were considered significant with a two-sided *p*-value of < 0.05

## Discussion

Despite tumor markers being easily accessible, their diagnostic ability for patients in unfavorable subsets had previously been unknown. Thus, we have evaluated tumor markers to identify patients in unfavorable subsets. ST439 and STN showed 100% specificity for patients in the unfavorable subset. No patients with elevated ST439 or STN above the reference value in the favorable subset were detected. In about 30% of the patients in the unfavorable subset, ST439 or STN was above the reference range. These results demonstrate that when ST439 or STN is elevated at the initial workup, a patient could be included in the unfavorable subset. In CUP treatment, the final diagnosis is not based soley on pathology, but on a combination of clinical factors. In addition, the standard of care for patients in unfavorable subsets is absent [3] and their prognosis is worse [1]. Therefore, it is necessary to differentiate between the favorable and unfavorable subsets within a limited time frame, such as one month [12]. Based on the findings from this study, routine evaluation of ST439 and STN could enable screening for treatment-ineffective subsets and prognostic estimation. This would enable identifying unfavorable subsets during the initial assessment for CUP. Refraining from aggressive treatments for patients in unfavorable subsets, who have a poor ECOG-PS, and early preparation for palliative care could improve the patients' quality of life. Evaluation of ST439 and STN at a patient’s first visit may help in the initial diagnosis of CUP in daily practice.

On the contrary, markers such as CA125, hCG, and PSA did not show a significant correlation between favorable and unfavorable subsets. This suggests that these markers are helpful when confirming favorable subsets with other clinical findings but are difficult to use alone for distinguishing between favorable and unfavorable subsets. The favorable subset included only five patients whose estimated primary organ was ovarian/peritoneal. There were no patients whose estimated primary organ was prostate or germ cell. A larger sample size is needed for further assessment. In addition, recent advances in histopathological examination of germ-cell tumors and malignant lymphomas suggest that anaplastic carcinoma of median development may not remain in a favorable subset as previously thought [27]. Therefore, the diagnostic abilities of tumor markers associated with malignant lymphomas (sIL-2R) and germ-cell tumors (hCG and AFP) may be limited given the current state of medicine.

An analysis comparing tumor markers and survival outcomes could not be carried out in this study because anticancer therapy was selected based on CUP histology or metastatic distribution and varied from patient to patient. We selected a primary site-directed treatment based on the suspected primary organ evaluated by a panel of oncologists. A previous report showed that patients with unfavorable subset CUP whose suspected primary organ was breast or ovary had higher response rates and a better prognosis compared with other unfavorable subsets [28].

This study has several limitations. It had a retrospective design and a relatively small sample size, with all data obtained from a single institution. In addition, the cut-off values were selected based on the facility standard. Whether these values are appropriate for distinguishing between favorable and unfavorable subsets of patients with CUP is unknown. Moreover, it is unclear why ST439 and STN can identify favorable or unfavorable subsets.

Additional research is needed regarding tumor markers that can identify favorable or unfavorable subsets in patients with CUP. Tumor markers can be utilized for the diagnosis of CUP in daily clinical practice.

## Conclusions

We evaluated diagnostic value of tumor markers in identifying favorable or unfavorable subsets in patients with CUP. ST439 and STN showed 100% specificity for the unfavorable subset. If ST439 or STN is elevated in patients with CUP, they could be included in the unfavorable subset.

## Data Availability

The datasets generated during and analyzed during the current study are not publicly available due to protect patient privacy but are available from the corresponding author on reasonable request.

## Abbreviations

AFP: α-Fetoprotein
CA125: Cancer antigen 125
CUP: Cancer of unknown primary
ECOG-PS: Eastern cooperative oncology group-performance status
hCG: Human chorionic gonadotropin
MRI: Magnetic resonance imaging
NA: Not applicable
NCCH: National Cancer Center Hospital
PIVKA-II: Protein induced by vitamin K absence or antagonist II
PSA: prostate-specific antigen
sIL-2R: Soluble interleukin-2 receptor
SLX: Sialyl Lewis X
ST439: NCC-ST 439
STN: Sialyl-Tn
